# Multi-lineage transcriptional and cell communication signatures define pathways in individuals at-risk for developing rheumatoid arthritis that initiate and perpetuate disease

**DOI:** 10.21203/rs.3.rs-6165802/v1

**Published:** 2025-03-31

**Authors:** Wei Wang, Cong Liu, Gary Firestein, Peter Skene, Kevin Deane, Michael Holers, Jane Buckner

## Abstract

Elevated anti-citrullinated protein antibodies (ACPA) levels in the peripheral blood are associated with an increased risk for developing rheumatoid arthritis (RA). Currently, no treatments are available that prevent progression to RA in these at-risk individuals. In addition, diverse pathogenic mechanisms underlying a common clinical phenotype in RA complicate therapy as no single agent is universally effective. We propose that a unifying set of transcription factor and their downstream pathways regulate a pro-inflammatory cell communication network, and that this network allows multiple cell types to serve as pathogenic drivers in at-risk individuals and in early RA. To test this hypothesis, we identified ACPA-positive at-risk individuals, patients with early ACPA-positive RA and matched controls. We measured single cell chromatin accessibility and transcriptomic profiles from their peripheral blood mononuclear cells. The datasets were then integrated to define key TF, as well as TF-regulated targets and pathways. A distinctive TF signature was enriched in early RA and at-risk individuals that involved key pathogenic mechanisms in RA, including SUMOylation, RUNX2, YAP1, NOTCH3, and β-Catenin Pathways. Interestingly, this signature was identified in multiple cell types, including T cells, B cells, and monocytes, and the pattern of cell type involvement varied among the at-risk and early RA participants, supporting our hypothesis. Similar patterns of individualized gene expression patterns and cell types were confirmed in single cell studies of RA synovium. Cell communication analysis provided biological validation that diverse lineages can deliver the same core set of pro-inflammatory mediators to receiver cells *in vivo* that subsequently orchestrate rheumatoid inflammation. These cell-type-specific signature pathways could explain the personalized pathogenesis of RA and contribute to the diversity of clinical responses to targeted therapies. Furthermore, these data could provide opportunities for stratifying individuals at-risk for RA, and selecting therapies tailored for prevention or treatment of RA. Overall, this study supports a new paradigm to understand how a common clinical phenotype could arise from diverse pathogenic mechanisms and demonstrates the relevance of peripheral blood cells to synovial disease.

### Introduction

Rheumatoid arthritis (RA) is a systemic immune-mediated disease marked by synovial inflammation and joint destruction[1]. Recent advances understanding its pathogenic mechanisms have led to novel treatments that markedly improved clinical outcomes, including targeted therapies that block cytokines or individual cell types such as B cells or T cells. Interestingly, responses to these agents are highly variable; a lack of response to one drug does not preclude a response to another with a different mechanism of action. These observations led us to propose that diverse mechanisms in individuals at risk for developing RA and with early RA converge to produce a common clinical phenotype. However, the divergent pathogenic pathways are poorly understood, and we currently lack reliable tests to predict benefit of targeted therapeutics for individual patients.

Current models suggest that seropositive RA begins with mucosal inflammation and loss of self-tolerance in individuals that carry certain genetic risk alleles and are exposed to risk-elevating environmental factors[2]. During a prolonged asymptomatic phase, circulating autoantibody levels increase, most notably anti-citrullinated protein antibodies (ACPAs) that are strongly associated with the future development of RA in up to 60% of at-risk individuals[3]. Several clinical trials have attempted to prevent onset of synovitis including treatment with atorvastatin, rituximab, methotrexate, hydroxychloroquine, and abatacept[4–8]. Although some interventions delayed conversion to clinical RA, only abatacept so far resulted in reduced rates of progression to RA during the trial period in ACPA-positive individuals.

These observations pose a challenging question: how do the heterogeneous mechanisms in at-risk individuals or early RA lead to a common phenotype? To address this, we formulated a hypothesis proposing that a unifying set of transcription factors and their downstream pathways regulate a pro-inflammatory cell communication network, and that this network enables multiple cell types to serve as pathogenic drivers in at-risk individuals or RA[1]. Thus, the clinical progression to and the phenotype of RA would be defined by a specific transcriptional program that orchestrates pro-inflammatory signals driving synovitis. Importantly, rheumatoid inflammation can arise from a diverse array of cell types in this model, which explains the variable response of RA and at-risk individuals to targeted therapies. This study is distinct from previous studies because it focused on defining pathways prior to onset of RA as opposed to longstanding established RA[9–11], which necessitated analysis of peripheral blood cells because synovial tissue is not accessible in at risk individuals.

To test this hypothesis and identify the pathways and cell types that predispose to developing RA, the Allen Institute for Immunology-UCSD-CU Transition to Rheumatoid Arthritis Project (ALTRA) identified at-risk individuals with elevated ACPAs. Along with early RA patients and controls, we evaluated peripheral blood mononuclear cells (PBMCs) using single cell technologies to define the transcriptome and chromatin accessibility. We then used a novel integrative analysis tool to determine whether there is a common set of drivers that can induce aberrant immunity. A distinctive TF signature was discovered that was enriched in peripheral blood immune cells of early RA and at-risk individuals. These signature TFs regulate key pathogenic processes in RA, including SUMOylation, RUNX2, YAP1, NOTCH3, and β-Catenin Pathways. Unexpectedly, this signature was identified in multiple cell types, including T cells, B cells, and monocytes, and the pattern of cell type involvement varied among the at-risk and early RA participants. We next biologically validated these predictions with cell-cell communication (CCC) analysis and found that any lineage displaying this RA TF signature can deliver overlapping sets of pro-inflammatory mediators to receiver cells *in vivo*. Their potential for orchestrating synovial inflammation was confirmed by demonstrating that the same genes are expressed by rheumatoid synovial cells. Importantly, composition of the signature cell types was highly variable between individuals. This diversity might contribute to highly variable clinical responses to targeted therapeutics in RA patients. Our approach holds promise for understanding distinct causal mechanisms in RA during the at-risk stage, improving risk stratification for future RA, and individualizing prevention and treatment strategies.

## Results

## Integrative single cell analysis reveals cell types in At-Risk/ERA and CON individuals

Peripheral blood mononuclear cells (PBMCs) were obtained from 26 ACPA positive (At-Risk) and 6 early RA (ERA) and 35 age and sex-matched controls (CON) and subjected to scATAC-seq and scRNA-seq (Fig. 1A, **Supplementary Table S1**). These data were used to assign each cell to a cell type with Latent Semantic Indexing (LSI) and Principal Component Analysis (PCA) to reduce the dimensionality of the scATAC-seq and scRNA-seq count matrices, respectively. Nearest neighbor graphs in reduced dimensions were built to identify clusters of cells. Uniform Manifold Approximation and Projection (UMAP) was then used to visualize the single cells in reduced dimension space (Fig. 1B). Both scRNA-seq and scATAC-seq cells were diffused evenly across the sample space, demonstrating a good integration across samples without batch effect (**Supplementary Fig. S1B, D**).

To integrate scRNA-seq and scATAC-seq for cell type, each cell in the scATAC-seq space was assigned a predicted gene expression profile from the cell in the scRNA-seq that was most similar. Cells from scRNA-seq and scATAC-seq were then clustered in the same co-embedding space for each sample (Fig. 1C). Each co-embedded cluster was treated as a pseudo-bulk cluster by summing gene counts from all the scRNA-seq cells and aggregating the raw scATAC-seq peaks. The annotation was defined by the cell type that occurs most frequently in the cluster. In total, 1610 pseudo-bulk clusters were retained in the final dataset, which included 703,701 scRNA-seq cells and 932,986 scATAC-seq cells, or 1,636,687 cells from 67 samples (median: 25194 cells/sample, 767 cells/cluster) after quality control (**Supplementary Table S2**).

The cells were assigned to 22 fine-grain transcriptional cell type for each sample (**Supplementary Table S3**). Thirteen major cell types, including B memory cells, B intermediate cells, B naive cells, CD14 monocytes (CD14 Mono), CD16 monocytes (CD16 Mono), CD4 naive T cells (CD4 T Naive), central memory CD4 T cells (CD4 TCM), CD8 naive T cells (CD8 T Naive), effector memory CD8 T cells (CD8 TEM), mucosal-associated invariant T cells (MAIT cells), natural killer cells (NK), CD56 bright natural killer cells (NK_CD56bright) and regulatory T cells (Treg), accounted for > 99% of total cells and had a sufficient number of cells for subsequent analysis (Fig. 1D). Two subtypes of CD4 T cells (CD4 T Naive and CD4 TCM) were the most abundant cell type among all 3 cohorts of PBMC samples with > 20% of total cells on average. B intermediate cells, B memory cells, CD16 Mono, NK_CD56bright, and Treg cells were relatively rare cell subsets with each comprising < 2% of total cells. The cell types showed similar distribution across At-Risk, ERA and CON groups except for B intermediate, B memory, and NK_CD56bright, which were modestly higher in At-Risk compared to two other groups (Centered Log-Ratio transformation followed by Kruskal-Wallis H test, p-value = 0.1, 0.04, and 0.08 respectively) (Fig. 1E). We then calculated the cluster purity as the percentage of the cells of most abundant cell type for all the 1610 clusters (**Supplementary Table S4**), which was 0.72 ± 0.19 across all clusters. The cluster purity showed minor different distributions across cell types (**Supplementary Fig. S1E**). B naive, CD14 Mono, CD16 Mono, MAIT, and NK displayed the highest purity scores (mean: 0.87 ± 0.13) while purity scores for T cell subsets were more diverse across clusters and relatively lower (mean: 0.68 ± 0.18). T cell subsets were sometimes included with other T cells. For instance, CD4 TCM cluster showed some other T cells like CD4 T Naive, CD8 T Naive, and CD8 TEM.

### Taiji analysis reveals distinctive TF patterns

Single cells within the same cluster are treated as one "pseudo-bulk" sample with the annotation as the cell type occurring most frequently in the cluster. The gene counts of scRNA-seq were added up and the fragments of scATAC-seq were combined to generate the RNA-seq input and ATAC-seq input for the pseudo-bulk samples respectively. We then applied the Taiji pipeline[12] to each individual cluster in each patient to evaluate the PageRank scores of TFs, which represents the importance of the TFs. Taiji has been experimentally validated in multiple biological contexts and demonstrated the robustness and reliability in revealing unappreciated roles of novel TFs in cell fate specification[13–15]. To characterize the global influences of all 1047 TFs across different pseudo-bulk clusters, we grouped the clusters based on the normalized PageRank across TFs. First, PCA was performed for dimension reduction of the TF score matrix with the first 500 principal components (PCs) retained for further analysis based on the "elbow" method, which explained 85% variance (**Supplementary Fig. S2A**). The first several PCs are primarily related to cell type rather than the disease state or the specific cohorts (**Supplementary Fig. S2B**). To determine the optimal number of groups and similarity metrics, Silhouette method was used to evaluate the clustering quality using five distance metrics: Euclidean distance, Manhattan distance, Kendall correlation, Pearson correlation, and Spearman correlation (**Supplementary Fig. S2C**). Pearson correlation was the most appropriate distance metric since the average Silhouette width is the highest among the five distance metrics.

We identified 5 Kmeans groups by unsupervised clustering, denoted G1 through G5, each of which showed distinct patterns of TF activity (**Supplementary Table S4**). The row-wise comparison demonstrates that some TFs have high PageRank scores in one or several Kmeans groups and suggests high TF activity in specific clusters (Fig. 2A; **Supplementary Fig. S2D**). In total, 640 TFs were identified as Kmeans group-specific TFs by comparing their PageRank scores between a specific group and the background groups (**Supplementary Table S5;** Fig. 2A). These TFs functionally correlated with assigned cell types. For instance, *KLF4*, which regulates monocyte differentiation[16], was G1-specific. G1 was enriched with two subsets of monocytes, including 59.5% CD14 Mono and 31.3% CD16 Mono. T-bet (encoded by *TBX21*) and *EOMES* displayed high activities in G3 where CD8 TEM and NK were the most abundant cell types with 37.9% and 40.3%, respectively. Those two genes are responsible for the cell fates of memory $CD8^+$ T cells and natural killer cells[17] (see Fig. 2A-B; **Supplementary Table S4** for lineage and group specific TFs that define each Kmeans group). Interestingly, more than half (409/640) of the TFs were G2-specific and their z scores were significantly higher in G2 compared to other groups. More than 80% (531/640) of the TFs were identified as key TFs for only one Kmeans group, suggesting the Kmeans groups had unique active TF patterns (**Supplementary Fig. S2E**).

### G2 is a multi-lineage group enriched with At-Risk/ERA and reveals an RA TF signature

The 5 Kmeans groups generally showed diverse compositions of cell types and disease states (**Supplementary Table S6-7**). As noted above, 4 of the 5 Kmeans groups had their own predominant cell types and accounted for more than 70% of their total clusters. G1, G3, G4, and G5 were enriched in monocytes; CD8 TEM and NK cells; CD4 T cells; B cells, respectively. However, G2 was unique in that it was mixed and displayed a cell type distribution similar to the overall PBMC distribution and included all 13 major cell types (Fig. 2B).

We then noted that G2 was significantly enriched in At-Risk and ERA clusters compared with CON (58% higher in At-Risk and ERA vs. CON, adjusted by the null distribution, p-value < 0.0001; Chi-squared test) and G4 was modestly enriched in CON clusters (24% higher in CON, p-value < 0.001; Chi-squared test) (Fig. 2C). Many interesting TFs were G2-specific, including zinc finger family members like *ZNF304*, *SP7*, *GLIS1*, *ZNF254*. For the subsequent analysis, we combined At-Risk and ERA (i.e., At-Risk/ERA) because their TF activity profiles and cell type distributions in G2 were nearly identical (p-value > 0.2; Wilcoxon rank-sum test). Moreover, the identified G2-specific TFs along with the enriched pathways for ERA and At-Risk respectively showed almost complete overlap (p-value < $10^{-5}$) (**Supplementary Fig. S2F-G**).

Multiple immunity-related TFs and the downstream genes regulated by those TFs conformed to pathways implicated in the pathogenesis of RA (Fig. 2D; **Supplementary notes**). This was particularly true for G2, where 5 relevant and significant pathways were identified, namely *SUMOylation of Intracellular Receptors*[18], *Transcriptional regulation by RUNX2*[19], *YAP1 and WWTR1-stimulated Gene Expression*[20], *NOTCH3 Intracellular Domain Regulates Transcription*[21], and *Deactivation of the β-Catenin Transactivating Complex*[22] Reactome pathways. The TFs and the representative target genes identified by our analysis are shown in **Supplementary Table S8**. These TFs and their downstream regulated genes are referred to as the *RA TF signature*. These TFs were significantly important in the signature pathways and the representative genes were among the top regulated genes by the corresponding TFs predicted by Taiji (**Methods**).

### The G2 RA TF signature is enriched in multiple cell types

Interestingly, we observed that the At-Risk/ERA TFs identified in G2 were present across all the major cell types analyzed (Fig. 3A), thereby establishing them as a hallmark “RA TF signature” and their downstream pathways as “signature pathways”. We further calculated the percentage of G2 clusters per cell type of total global clusters for At-Risk/ERA and CON groups (Fig. 3B; **Supplementary Fig. S2H**). Notably, CD4 T Naive, CD4 TCM, and CD8 T Naive showed the greatest enrichment in At-Risk/ERA compared to CON (31% vs 18%, p-value < 0.01; 23% vs 12%, p-value < 0.01; 65% vs 26%, p-value < 0.01, respectively for At-Risk/ERA compared with CON; Chi-squared test). Of interest, MAIT cells with the TF profile were only found in CON clusters (0% vs 43% for At-Risk/ERA and CON, p-value < 0.1; Chi-squared test). Despite the negative correlation between MAIT cell abundance and age, the comparable age of the CON group with At-Risk/ERA (**Supplementary Table S1**) suggests that age does not account for these differences and MAIT cells might be protective of conversion/progression of RA. Overall, the top RA signature TFs determined by unsupervised clustering showed significantly higher PageRank scores in G2 compared to other groups across all cell types (Fig. 3C).

All the major cell types were enriched in this common set of At-Risk/ERA signature pathways while some individual cell types demonstrated specific enriched pathways (Fig. 3D). For example, activation of HOX genes was enriched in B cells, CD4 T cells, CD8 T Naive, and monocytes. RUNX3 regulation is more highly associated with CD8 TEM, NK, CD4 T Naive, and monocytes[23]. Despite individual variations described above, the general pattern of pathways associated with pathogenesis of RA is consistent and extends across the identified cell types.

### Patterns of cell types with the G2 RA TF signature are highly variable across individuals

We then determined which cell types display the TF signature in each member of the At-Risk and ERA cohorts. Multiple combinations of cell types were identified in individual participants (Fig. 3E). Twenty-five out of 26 At-Risk and all 6 ERA participants had the signature in at least one cluster and in at least one of the key cell types. The one negative At-Risk individual could have had a similar pattern in a rare cell type beyond the resolution of this analysis. However, the distribution of cell types was highly variable among participants. In some cases, only one cell type was identified for an individual participant, while in others there were multiple cell types displaying the pattern. For instance, participant 9 had clusters with the signature in all the cell types except NK and Treg, while participant 27 only had CD4 TCM clusters. Some patients displayed more even distribution across multiple cell types like participant 31 while others had predominant signature cell type like participant 3.

Among all the involved cell types, the signature was most enriched in T cell types including CD4 T Naive, CD8 T Naive, CD4 TCM, and CD8 TEM (Fig. 3E). Different cell types also displayed diverse distribution patterns across patients. CD4 Naive and CD4 TCM had much wider appearances in many patients while Treg, B cell and monocytes were only found in a few participants. Therefore, the patterns displayed by various individuals were diverse with highly variable cell types. Some CONs also displayed these signatures although the number of clusters was significantly less than At-Risk/ERA, particularly for certain T cell subsets ($p$-value $< 0.005$; Wilcoxon rank-sum test) (**Supplementary Fig. S3A**).

## Distinct cellular communication networks in At-Risk/ERA and control participants

After demonstrating individualized patterns of signature cluster cell types in At-Risk/ERA, we then investigated how the signature cells communicate to determine how inflammation signals are transmitted. Cell-cell communications (CCC) were analyzed by correlating expression levels of ligands such as cytokines in the source cells with their corresponding receptor expressions in the receiver cells for each individual using CellChat[24]. To compare At-Risk/ERA and CON groups, we first aggregated CCC between the same signature cells across all the ligand-receptor pairs and all the individuals within the group. We observed distinct CCC patterns: At-Risk/ERA participants displayed significantly more interactions within signature clusters than controls, particularly between T cells and NK cells. Cellular communications with signature monocytes were less common and only observed in the At-Risk/ERA group (Fig. 4A). The difference between the total number of CCC in the two groups was approached statistically significant (p-value = 0.06 using Wilcoxon rank-sum test).

We next evaluated the cellular communication strength. Notably, communication between CD8 T Naive and CD4 TCM were more pronounced in At-Risk/ERA group, while communications between CD4 T Naive, and CD8 TEM were more intense in controls (Fig. 4B). The total communication strength in At-Risk/ERA was significantly higher than control group (p-value = 0.04 using Wilcoxon rank-sum test). As a representative example, participant 53 from control group and participant 9 from At-Risk/ERA group had the most diverse cell type distribution in signature clusters (**Supplementary Fig. S3A;** Fig. 4C), providing an overview of almost all the cell types. It is worth noting that the number and intensity of the total CCC aggregating all the clusters from all the Kmeans groups were comparable between the At-Risk/ERA and CON groups, highlighting the importance of the signature cells differentiating the two groups (**Supplementary Fig. S3E**).

### Biological validation of RA TF signature cells effect on pathogenic cells

A diverse array of inflammatory cytokines, chemokines, and growth factors contribute to a core set of inflammatory mediators that have been implicated in RA pathogenesis. We curated a list of these mediators (**Methods; Supplementary Table S9**), collectively referred to as “pathogenic genes”. As with the diversity of signature cell types across individuals, the CCC pattern transmitting the inflammatory signals also varied from individual to individual. For example, major senders and receivers were variable among individual participants (**Supplementary Fig. S3F**). Some individuals such as participant 5, 26, and 27 used only one cell type as major communicator while others like participant 9, 18, and 23 relied on multiple cell types. Among those with multiple cell types, some displayed more even distributions of signals across cell types like participant 9 and 23 while others exhibited a predominant signature cell type (e.g., CD8 TEM in participant 18). Importantly, the mRNA transcripts of receiver cells validate the predicted in vivo response to these inflammatory signals regardless of their source, highlighting the agnostic nature of cellular responses to the inflammatory mediators.

Out of the identified significant ligand-receptor pairs in each participant, twelve ligand-receptor pairs were related to this pathogenic gene set. We ranked the important pathways based on the difference in total information flow within signature clusters when comparing At-Risk/ERA to control samples. The

IL16 - CD4, CD160 - TNFRSF14, TGF-β1 - (TGFBR1 + TGFBR2), and BTLA - TNFRSF14 were the most prominent ligand-receptor pairs enriched in At-Risk/ERA considering both the difference and absolute information flow values (Fig. 4D).

The IL16 - CD4 signaling pathway, which has been implicated in RA[25], showed significantly stronger signals in At-Risk/ERA group than control group. For instance, participant 31 from At-Risk/ERA group and participant 48 from control group have similar cell type distribution in signature clusters (**Supplementary Fig. S3A**). Participant 31 displayed denser and stronger interactions than participant 48 and signature clusters are more likely to act as major senders than receivers (Fig. 4E). The outgoing and incoming signal patterns of IL16 - CD4 pair demonstrated the diverse nature of sender cells and the agnostic response of receiver cells (Fig. 4F). Multiple cell types send signals of IL-16, including B cells and monocytes that are unique senders in At-Risk/ERA and CD8 TEM and monocytes are unique receivers, responding to IL-16 signals regardless of their source. CD4 T cells are most widely used as communicators across participants while B cells and NK cells only act as senders in IL-16 signaling pathway.

Other interesting and relevant pro-inflammatory pathways were also enriched in At-Risk/ERAs. For instance, TGF-β1, which is an important regulator in RA, showed much denser and stronger intercellular communications in participant 18 from At-Risk/ERA than participant 48 from control group (**Supplementary Fig. S4A**). Signature clusters were more likely to act as major senders in TGF-β1 signaling pathway, particularly in CD4 T cells and CD8 TEM cells (**Supplementary Fig. S4B**). On the other hand, signature clusters mainly acted as major receivers in CD160 - TNFRSF14 signaling pair (**Supplementary Fig. S4C-D**). NK cells were the most widely used cell type in CD160 signal communication. In BTLA – TNFRSF14 signaling, B cells are the only cell type acting as senders while multiple cell types are the receivers (**Supplementary Fig. S4E-F**). These expression patterns confirm the prediction that cells within At-Risk RA microenvironment had been exposed to and were actively responding to inflammatory mediators, regardless of the sender cellular origin.

We then developed a random forest classification model with pathogenic gene expression as features. Sixty-three genes were identified as candidate predictors, which were active across each At-Risk/ERA participant in signature group G2 (**Methods**). The test accuracy was monotonically increasing with more predictors, reaching a plateau of 0.93 (**Supplementary Fig. S5A**). Top predictors included *MMP23B, TGFB1, IFNL1, CCL5*, and *IL15* (Fig. 5A). *MMP23B*, which emerged as a top predictor in classification model, showed elevated gene expression level in At-Risk/ERA compared to control (Fig. 5B). *MMP23B* plays a role in regulating the Kv1.3 potassium channel, which has been implicated in autoimmunity[26]. *TGFB1* also showed elevated gene expression in At-Risk/ERA (Fig. 5C). Additionally, we explored the relationship between the G2 RA signature TFs identified above and *TGFB1* as their target gene. The signature regulators of *TGFB1* included some well-known RA-related TFs like *RORC, TFAP2A*, and *KLF1* (Fig. 5D).

Gene expression was greater for the top 30 predictors in At-Risk/ERA participants compared with controls, including *CCL4*, *IL12A*, *TNFSF14*, *IL15*, *NOTCH1*, and *CCL5* (Fig. 5E). To validate our predictions, we assessed protein expression levels of 6 genes using proteomics, each of which confirmed significant increased protein expression level in the serum of At-Risk/ERA group compared to controls (CCL3, CCL4, IFN-λ1, IL-15, TGF-β1, and TNFSF14) (Fig. 5F).

Although a common set of pathogenic genes were shared across At-Risk/ERA participants, the cell types that were most likely to produce the specific gene were highly variable (**Supplementary Fig. S5B**). For instance, the top 5 predictors were active in CD8 TEM cells and NK cells in most participants while a few participants expressed the genes through CD4 TCM, and CD8 T Naive cells. *NOTCH1* and *CXCL16* displayed uniform activity across all cell types with the highest activity in Tregs. Some genes showed exclusively high activity in specific cell types, such as *TNFSF9* in CD4 TCM and *ADAMTSL4* in monocytes. Mediator expression patterns were individualized towards specific cell types. For example, *TGFB1* was highly expressed in CD8 TEM and NK cells in most of the patient while it was more highly expressed in B cells in participant 13 and monocytes in participant 7 (**Supplementary Fig. S5C**). These findings suggest that At-Risk/ERA individuals express a common set of pathogenic genes, driven by any cell type possessing RA TF signature.

We then evaluated the Accelerating Medicine Partnerships (AMP) synovial scRNA-seq data to determine if the RA gene expression signature observed in peripheral blood was confirmed in the inflamed tissue and whether a diversity of cell types was also present[11]. As further biologic confirmation of the *in vivo* relevance of our peripheral blood cell observations, the top mediators were also found in synovial cells and, more strikingly, there was broad diversity of the cell types within the synovial tissues that expressed them (Fig. 6A). The number of genes expressed across all cell types displayed distinct patterns across samples (Fig. 6B). This heterogeneity suggests that each RA patient, like pre-RA and early RA PBMCs, have this molecular signature. We then determined which cell types display the regulator signature for each patient. As with PBMCs, distribution of cell types was highly variable among RA samples (Fig. 6C). In some cases, one dominant cell type was identified for an individual participant, such as monocytes in sample BRI-456, while in others there were multiple cell types, such as instance, BRI-552, displayed expression across all the cell types.

## Discussion

Our study provides compelling evidence that individuals with those at elevated risk for developing RA and even early RA exhibit consistent TF signatures in peripheral blood immune cells. These signatures, which involve pathways implicated in disease pathogenesis, could contribute to disease onset and persist after transition to classifiable RA. The signature TFs regulate key genes in pathways like *SUMOylation*, *RUNX2*, *YAP1*, *NOTCH3*, and *β-Catenin* Pathways, each of which has been implicated in RA pathogenesis. Serum protein levels for key genes in the TF pathways were also elevated and provide biologic confirmation of the transcriptome data. These predictions were biologically validated by demonstrating appropriate receiver cell responses *in vivo* as well as similar gene expression patterns in RA synovial tissue cells.

Perhaps the most intriguing observation is that the signatures occurred in multiple cell types, each of which would have arthritogenic potential. The signature TFs drive a defined set of pro-inflammatory genes that, in turn, could contribute to the onset and eventually the perpetuation of RA. By employing classification models, we defined candidate genes that could orchestrate the transition process to clinical synovitis including *TGFB1*, *MMP23B*, *IL16*, and *TNFSF14*, which were confirmed by protein expression data. Thus, the common drivers regulated by this RA TF signature and the signals transmitted through the expression and release of a diverse array of inflammatory mediators in a receptive environment could trigger disease transitions. Analysis on cellular signaling network further confirmed that the responding cells responsible for transition are actively transcribing the appropriate genes and are agnostic about which cell provides the signal as long as the receiving pathogenic cell has access to them.

While the blood RA TF signature is observed in all cell types at the group level, there is considerable heterogeneity related to the specific cell types implicated among individuals. One of the most provocative findings was the individualized nature of cell types in each at-risk and ERA patient. This suggests a common driver in multiple cell types as suggested above, although it is not uniform across participants or cell states. Each participant exhibits a distinct combination of cell types, along with unique subsets of signature pathways and pathogenic genes, suggesting a significant stochastic component to remodeling the epigenome. In addition, the relative importance of individual TFs and pathways varied among cell types and participants. This phenomenon implies distinct pathogenic processes in individuals that relate to which cell types are involved and promote progression from pre-RA to RA. Moreover, they could potentially contribute to the diversity of clinical responses to targeted agents in RA[1,27–29]. In other words, the clinical responses could depend on which cell types express the pathogenic genes, such as B cells (rituximab) or CD4 T cells (abatacept) or the specific signal received by the receiver cells (e.g., *TGFB1* or *IL16*).

Our study was unique in that it integrated transcriptome and chromatin accessibility data to reveal pathways that would have been missed by transcriptome-only analysis[30]. In addition, the RA TF signature was identified by clustering TF PageRank scores calculated by Taiji from pseudo-bulk clusters in each individual participant. This approach is distinct from single cell analysis that typically aims to identify cell clusters unique to disease compared to control. Such a single cell analysis would not enable discovery of the signature given the high variability of signature TFs and cell types in individual participants.

While previous studies have predominantly focused on established RA synovium or the transcriptome peripheral blood in at-risk individuals[9–11,31,32], our study explored and integrated transcriptome and chromatin accessibility in PBMCs from at-risk individuals. Perhaps most interesting, many of the pathways and genes that we discovered in at-risk individuals have also been observed in synovial tissue cells, especially within certain T cell clusters. For instance, CCL5, identified as a key player in both communication pathway and a top pathogenic gene in our study, is also a top maker gene of CD8 + GZMK + memory clusters in RA synovial tissues[9,11]. Our findings also corroborate previous research in highlighting the importance of several other chemokines including CCL4, CCL4L2, CCL3, XCL1, and XCL2. Furthermore, TNFSF9 and IFNG, which emerged as top predictors in our model, are also noted in CD4 T cells isolated from established RA synovium[11]. The concordance between our pre-RA PBMC and established RA synovium data suggest that blood sampling is relevant to synovial mechanisms and that potential early biomarkers or patient stratification is feasible using PBMCs.

The primary findings in other analyses of peripheral blood cells in at-risk individuals focused on CD4 + T naïve cells or CCR2 + CD4 + T cells[32,33]. However, a preponderance of a single pathogenic cell type would not explain the diversity of responses to targeted agents like abatacept or even anti-CD4 antibodies[34]. The same cell types are identified in our analysis, but many others were also identified based on the RA TF signature. The ability to discover other potentially pathogenic cells is likely due to the greater resolution afforded by integration of transcriptome and chromatin accessibility and discovering the most relevant TFs. This method also allows identification of distinct patterns of pathogenic cell types for each participant. This improved resolution confirms our previous observation that combining both technologies markedly increases the ability to distinguish between cell populations and pathways[30]. CD4 + T cells certainly account for many of the clusters in our analysis, but B cells, CD8 + T cells, monocytes and NK cells can also exhibit the signature and produce the same pathogenic mediators as CD4 + T cells in some participants. The expanded repertoire of disease-associated cells likely contributes to variable mechanisms of RA.

Participant-specific patterns of pathogenic cell types were discovered not only in peripheral blood cells in at-risk individuals and early RA patients, but also in synovial tissues in RA patients. Each RA patient has an individualized pattern of cell types expressing the top pathogenic mediators. Taken together, these findings support our hypothesis on the contribution of multiple cell types to a common clinical phenotype, leading to the variable efficacy of specific anti-rheumatic agents. These insights pave the way for determining biomarkers that might predict who will progress from at-risk to classifiable disease, developing mechanism-based prevention strategies in pre-RA, and using signatures to pursue individualized treatment approaches in established RA.

The surprising broad overlap of the RA TF signature, genes, and pathways across multiple cell types suggests that there might be common mechanisms that shape the RA-associated transcriptome and epigenome. The nature of these influences is not yet known, but its consistency across the spectrum of cell types suggests that they are shared. Environmental and mucosal stresses, especially in the airway due to its critical role in the RA, are possible influences because all circulating cell types can be exposed to irritants at these sites. For example, cigarette smoke is a known risk factor for RA and can induce stress throughout the airway. Smoking is also associated with alterations in the epigenome of peripheral blood cells[35]. We also previously described shared DNA methylation abnormalities in circulating B cells and memory and naive CD4 T cells in the at-risk population[36], which supports this concept. It is also possible that multiple cell types in G2 are influenced by similar inflammatory signals, but the impact could be divergent depending on where they are imprinted (e.g., gut, lung, or synovium).

Our study primarily focused on pre-RA, but we also observed similar patterns in early RA. However, it remains uncertain whether the signature is specific to pre-RA due to the absence of comparable datasets for other "at-risk" populations, such as those predisposed to systemic lupus erythematosus or inflammatory bowel disease. It is plausible that this signature represents a general phenomenon occurring during the "at-risk" period across various immune-mediated diseases. If so, the ultimate manifestation of a particular autoimmune disease might be determined by other factors, such as genetic predisposition and environmental influences. This phenomenon could provide insight into the variability in therapeutic responses observed across different diseases. Nevertheless, in certain instances, this scenario seems unlikely. For instance, the majority of psoriasis patients respond favorably to Th17-directed therapies[37], suggesting a more limited cellular repertoire driving disease pathology compared to RA. Thus, while the immune signature identified in pre-RA may have broader relevance, its specificity and cellular distribution likely vary across autoimmune diseases, warranting further investigation.

In conclusion, our study defined a distinctive RA TF signature and genes enriched in the peripheral blood mononuclear cells at-risk individuals and early RA. These TFs are involved in the known pathogenic pathways, offering insights into the molecular events that lead to RA. Analysis of cell-cell communication shows that the signature-bearing cells deliver shared pro-inflammatory signal to receiver cells. Notably, the signatures are present in diverse cell types from different individuals, providing a potential explanation for the diverse clinical responses with targeted therapeutics. We propose that multiple cell types can be responsible for the transition to clinical arthritis, yet the receiver cells do not discriminate regarding the source of the signal. These individualized signature patterns potentially open avenues for prognostic tests and personalized treatments. Overall, our findings represent a novel paradigm for understanding how a common clinical phenotype arises from diverse mechanisms (Fig. 6D). Similar processes might account for variable therapeutic responses in other immune-mediated diseases.

### Materials and Methods

## Clinical cohorts

Three groups of participants were recruited for this study. The demographics and baseline characteristics of the cohorts are provided in **Supplementary Table S1**. The first cohort (At-Risk) included individuals who were at-risk for future clinical RA as indicated by serum ACPA positivity > 2x the upper limit of normal[2] using the assay anti-cyclic citrullinated peptide-3 anti-CCP3, IgG ELISA (Werfen, San Diego, CA USA). The second cohort (ERA) was comprised of patients who were anti-CCP3 positive and had early RA meeting the 2010 American College of Rheumatology/European Alliance of Associations for Rheumatology (ACR/EULAR) classification criteria for RA and were diagnosed < 1 year from study enrollment[37]. The At-Risk and ERA participants were identified and recruited at the University of Colorado

Anschutz and UC San Diego. The third cohort (Controls) was comprised of participants without inflammatory arthritis who were recruited at the Benaroya Research Institute and the University of Colorado Anschutz. The studies were approved by ethical review boards at the University of Colorado Anschutz, UC San Diego and the Benaroya Research Institute, and all participants gave informed consent.

## Genomic data acquisition

Sample preparation

Blood was drawn into BD NaHeparin vacutainer tubes (for PBMC; BD #367874) or K2-EDTA vacutainer tubes (for plasma; BD #367863). PBMC isolation and plasma processing were started within 2 hours post draw. For PBMC isolation, the samples in NaHeparin tubes for each donor were pooled into one common pool and combined with an equivalent volume of room temperature PBS (ThermoFisher #14190235). PBMCs were isolated using Leucosep tubes (Greiner Bio-One #227290) with 15 ml of Ficoll Premium (GE Healthcare #17-5442-03). After centrifugation, the PBMCs were recovered and resuspended with 15 ml cold PBS + 0.2% BSA (Sigma #A9576; “PBS + BSA”). The cells were pelleted, resuspended in 1 ml cold PBS + BSA per 15 ml whole blood processed and counted with a Cellometer Spectrum (Nexcelom) using Acridine Orange/Propidium Iodide solution. PBMCs were cryopreserved in 90% FBS (ThermoFisher #10438026) / 10% DMSO (Fisher Scientific #D12345) at a target of $5 \times 10^6$ cells/ml by slow freezing in a Coolcell LX (VWR #75779-720) overnight in a -80°C freezer followed by transfer to liquid nitrogen.

For genomics assays PBMCs were removed from liquid nitrogen storage and immediately thawed in a 37°C water bath. Cells were diluted dropwise into 40 mL AIM V media (Thermo Fisher Scientific #12055091) pre-warmed to 37°C. Cells were pelleted at 400 x g, resuspended in 5 mL cold AIM V media, and recounted using a Cellometer Spectrum. 30 mL cold AIM V media was added to the cells, which were re-pelleted and resuspended to appropriate concentration for the assays.

scRNA-seq scRNA-seq was performed on PBMCs as previously described[38] *(P. C. Genge, STAR Protoc 2, 100900 (2021))*. In brief, scRNA-seq libraries were generated using a modified 10x genomics chromium 3' single cell gene expression assay with Cell Hashing. Sample libraries were constructed across different batches, with the addition of a common control donor leukopak sample in each library as batch control. Libraries were sequenced on the Illumina Novaseq platform. Hashed 10x Genomics scRNA-seq data processing was carried out using BarWare[39] to generate sample-specific output files.

scATAC-seq

## FACS neutrophil depletion

To remove dead cells, debris, and neutrophils prior to scATAC-seq, PBMC samples were sorted by fluorescence-activated cell sorting (FACS) following established protocols[38]. Cells were incubated with Fixable Viability Stain 510 (BD, 564406) for 15 minutes at room temperature and washed with AIM V medium (Gibco, 12055091) before incubating with TruStain FcX (BioLegend, 422302) for 5 minutes on ice, followed by staining with mouse anti-human CD45 FITC (BioLegend, 304038) and mouse anti-human CD15 PE (BD, 562371) antibodies for 20 minutes on ice. After washing, cells were then sorted on a BD FACSAria Fusion with a standard viable CD45 + cell gating scheme. Neutrophils were then excluded in the final sort gate. An aliquot of each post-sort population was used to collect 50,000 events to assess post-sort purity.

## Sample processing

Permeabilized-cell scATAC-seq was performed as described previously[38]..A 5% w/v digitonin stock was prepared stored at – 20°C. To permeabilize, $1×10^6$ cells were centrifuged and resuspended in cold isotonic Permeabilization Buffer. Then they were diluted with 1 mL of isotonic Wash Buffer and centrifuged, and the supernatant was slowly removed. Cells were resuspended in chilled TD1 buffer (Illumina, 15027866) to a target concentration of 2,300 – 10,000 cells per µL. Cells were filtered through 35 µm Falcon Cell Strainers (Corning, 352235) before counting on a Cellometer Spectrum Cell Counter (Nexcelom) using ViaStain acridine orange/propidium iodide solution (Nexcelom, C52-0106-5).

## Sequencing library preparation scATAC-seq libraries were prepared following established protocol[38]. In brief, 15,000 cells were combined with TD1 buffer (Illumina, 15027866) and Illumina TDE1 Tn5 transposase (Illumina, 15027916) and incubated at 37°C for 60 minutes. A Chromium NextGEM Chip H (10x Genomics, 2000180) was loaded and a master mix was then added to each sample well. Chromium Single Cell ATAC Gel Beads v1.1 (10x Genomics, 2000210) were loaded into the chip, along with Partitioning Oil. The chip was loaded into a Chromium Single Cell Controller instrument (10x Genomics, 120270) for GEM generation. After the run, GEMs were collected and linear amplification was performed on a C1000 Touch thermal cycler.

GEMs were separated into a biphasic mixture with Recovery Agent (10x Genomics, 220016), and the aqueous phase was retained and removed of barcoding reagents using Dynabead MyOne SILANE and SPRIselect reagent bead clean-ups. Sequencing libraries were constructed as described in the 10x scATAC User Guide. Amplification was performed in a C1000 Touch thermal cycler. Final libraries were prepared using a dual-sided SPRIselect size-selection cleanup.

## Quantification and sequencing

Final libraries were quantified using a Quant-iT PicoGreen dsDNA Assay Kit (Thermo Fisher Scientific, P7589) on a SpectraMax iD3 (Molecular Devices). Library quality and average fragment size were assessed using a Bioanalyzer (Agilent, G2939A) High Sensitivity DNA chip (Agilent, 5067 – 4626).

Libraries were sequenced on the Illumina NovaSeq platform with the following read lengths: 51nt read 1, 8nt i7 index, 16nt i5 index, 51nt read 2.

Plasma proteomics

Plasma samples were run on the Olink Explore 1536 platform. Analytes from the inflammation, oncology, cardiometabolic, and neurology panels were measured. Samples were randomized across plates to achieve a balanced distribution of age and sex. Resulting data were first normalized to an extension control that was included in each sample well. Plates were then standardized by normalizing to inter-plate controls run in triplicate on each plate. Data were then intensity normalized across all samples. Final normalized relative protein quantities were reported as log2 normalized protein expression (NPX) values by Olink. Three protein analytes were repeated across each of the four panels and treated as distinct measurements: TNF, IL-6, and CXCL8. Data, including QC flags, were reviewed for overall quality prior to analysis. Samples were measured across multiple batches.

To facilitate comparisons between batches, plasma from 12 donors was obtained commercially (BioIVT; Bloodworks Northwest) and randomly interspersed among the above study samples. Samples measured in later batches were bridge normalized to the earliest batch. Bridge offsets were determined for each batch and each analyte separately by taking the median of the per-sample NPX differences between the later batch result and the earliest (reference) batch result for the 12 commercial samples. Offsets were then subtracted from the analyte measurements of all samples in the later batch to obtain the normalized NPX values.

## Dataset integration

Paired scRNA-seq and scATAC-seq datasets from each participant were obtained from 26 At-Risk individuals with elevated anti-citrullinated protein antibody (ACPA), 6 seropositive ERA patients and 35 controls (CON). The detailed clinical information is summarized in **Supplementary Table S1**.

*10x scRNA-seq data.* scRNA-seq data were aligned using 10x cellranger v3.1.0 and 10x transcriptome vGRCh38-3.0.0. Hashtag Oligo sequences were processed using CITE-Seq Count v1.4.3, and cells were assigned to sample-linked hashes, split by sample for each well, and merged across wells per sample using an AIFI pipeline. Cells were labeled using Seurat v4 labeling pipeline with default parameters. The reference was customized based on the recently described CITE-seq reference of 162,000 PBMC measured with 228 antibodies[40]. QC summary plots along with statistics can be found in **Supplementary Fig. S1A** and **Supplementary Table S2**,

*10x scATAC-seq data.* In the scATAC-seq pipeline, we implemented CellRanger alignment, followed by a rigorous quality control process. We retained cells with unique fragments between 1000 and 100,000, fragment size between 10 and 2000, > 50% of fragments in Altius, > 20% of fragments in transcription starting site (TSS), > 4 TSS enrichment score. This ensures that cells from the scATAC-seq pipeline are high quality, reduces the number of doublets, and are available in a variety of formats for downstream analysis (.arrow, fragments.tsv.gz, and .h5-formatted count matrices). scATAC-seq data were aligned using 10x cellranger-atac v1.1.0, using reference vGRCh38-1.1.0. After alignment, data were processed through a custom QC and counting pipeline to generate a matrix of unique fragment counts in each peak. ArchR v1.0.2 was used to generate Arrow files, doublet filtering (filterRatio = 0.5), dimensionality reduction with iterative latent semantic indexing (LSI) (iterations = 4), and clustering (resolution = 3). QC summary plots along with statistics can be found in **Supplementary Fig. S1C** and **Supplementary Table S2**,

*Integration of scRNA-seq and scATAC-seq data.* scATAC-seq data were integrated with the corresponding scRNA-seq using the "addGeneIntegrationMatrix" function in ArchR with default parameters. After alignment, each cell in the scATAC-seq space was assigned a gene expression signature from the cell in the scRNA-seq that is the most similar. Cells from both scRNA-seq and scATAC-seq were clustered in the same co-embedding space.

## TF regulatory networks construction based on Taiji

Single cells within the same cluster were treated as one "pseudo-bulk" sample with the annotation as the cell type occurring most frequently in the cluster. The gene counts of scRNA-seq were added up and the fragments of scATAC-seq were combined to generate the RNA-seq input and ATAC-seq input for the pseudo-bulk samples respectively. Only pseudo-bulk samples with > 2000 open chromatin peaks, > 20 scATAC-seq cells and > 20 scRNA-seq cells were kept on account of reliability of constructed regulatory networks. Additionally, to link promoters and enhancers, the promoter-enhancer contacts predicted by Epitensor v0.9 was used. Taiji v1.1.0 with default parameters was used for the integrative analysis of RNA-seq and ATAC-seq data. The motif file was downloaded directly from the CIS-BP database containing 1078 human motifs.

## Taiji pipeline overview

To characterize TF activity in each pseudo-bulk cluster, we performed an integrated multi-omics analysis using the Taiji pipeline[12,15]. Taiji integrates gene expression and epigenetic modification data to build gene regulatory networks. The algorithm first predicts putative TF binding sites in each open chromatin region that mark active promoters and enhancers using motifs documented in the CIS-BP database[41]. These TFs are then linked to their target genes predicted by EpiTensor[42]. The regulatory interactions are assembled into a genetic network. Finally, the personalized PageRank algorithm is used to assess the global influences of the TFs. In the network, the node weights are determined by the z scores of gene expression levels, allocating higher ranks to the TFs that regulate more differentially expressed genes. Each edge weight is set to be proportional to the TF's expression level, its binding site's open chromatin peak intensity, and the motif binding affinity, thus representing the regulatory strength. Using this method, Taiji has more power than other methods that identify key regulators in individual transcriptome and chromatin accessibility and has been confirmed using simulated data, literature evidence and experimental validation in numerous studies of various biological problems[12–15]. For this dataset, the median number of nodes and edges of the networks were 17,046 and 3,002,662, respectively, including

1047 (6.14%) TF nodes. On average, each TF regulates 3417 genes, and each gene is regulated by 184 TFs.

## TF regulatory networks weighting scheme

As described in the original Taiji paper[12], a personalized PageRank algorithm was applied to calculate the ranking scores for TFs. We first initialized the edge weights and node weights in the network. The node weight was calculated as $e^{z_i}$, where $z_i$ is the gene's relative expression level in cell type $i$, which is computed by applying the $z$ score transformation to its absolute expression levels. The edge weight was determined by $e_{ij} = \sqrt{g\sum_{k=1}^{n} p_k * m_k}$, where $p$ is the peak intensity, calculated as $\frac{1}{1+e^{-(x-5)}}$, where x is $-log_{10}(p)$, represented by the p-value of the ATAC-seq peak at the predicted TF binding site, rescaled to [0, 1] by a sigmoid function; $m$ is the motif binding affinity, represented by the p-value of the motif binding score, rescaled to [0, 1] by a sigmoid function; $g$ is the TF expression value; $n$ is the number of binding sites linked to gene $j$. Let s be the vector containing node weights and W be the edge weight matrix. The personalized PageRank score vector v was calculated by solving a system of linear equations $v = (1 - d)s + dWv$, where d is the damping factor (default to 0.85). The above equation can be solved in an iterative fashion, i.e., setting $v_{t+1} = (1-d)s + dWv_t$.

If the TFs in the same protein family share the same motifs, their PageRank scores are distinguished by their own expression levels because their motifs and the target genes are the same. If a motif is weak, the PageRank score of the TF is decided by whether these motifs occur in the open chromatin regions (measured by the peak intensity of the ATAC-seq data), the TF expression and its target expression levels. The relative difference between the PageRank scores of TFs also helps to uncover important TFs with weak motifs.

## Unsupervised clustering analysis

To identify the groups of samples showing similar TF activity profile, we clustered the samples based on the normalized PageRank across TFs. First of all, we performed the principal component analysis (PCA) for dimension reduction of the TF score matrix. We retained the first 500 principal components (PCs) for further clustering analysis based on "elbow" method, which explained 85% variance (**Supplementary Fig. S2B**). To find the optimal number of groups and similarity metric, we performed the Silhouette analysis to evaluate the clustering quality using five distance metrics: Euclidean distance, Manhattan distance, Kendall correlation, Pearson correlation, and Spearman correlation (**Supplementary Fig. S2C**). Pearson correlation was the most appropriate distance metric since the average Silhouette width was the highest among the five distance metrics. Based on these analyses, we identified 5 Kmeans groups showing distinct dynamic patterns of TF activity.

## Identification of Kmeans group-specific TFs

To identify Kmeans group-specific TFs, we divided the clusters into two groups: target group and background group. Target group included the clusters in the Kmeans group of interest and the background group comprised the remaining clusters. We then performed the normality test using Shapiro-Wilk's method to determine whether the two groups were normally distributed and we found that the PageRank scores of most clusters (95%) didn't follow normal or log-normal distribution. Thus, Mann-Whitney U Test was used to calculate the P-value. Double cutoffs, i.e. P-value ≤ 0.01 and log2 fold change ≥ 0.5, were used for calling specific TFs. Results were summarized in **Supplementary Table S5**.

## TF regulatee analysis

Taiji generated the regulatory network file for each cluster showing the regulatory relationship between TF and regulatees with edge weight, which represents the regulatory strength. Regulatees in **Supplementary Fig. S3B,C** are top 500 regulatees ranked by mean edge weight across G2-specific TFs. Representative regulatees in **Supplementary Table S8** were selected as the top 10 genes regulated by the signature TFs involved in each pathway ranked by the mean edge weight.

## Pathway enrichment analysis

The enriched functional terms in this study were analyzed by R package clusterProfiler_4.0.5. A cutoff of P-value ≤ 0.05 was used to select the significantly enriched Reactome pathways.

## Cell-cell communication analysis

The R package CellChat_2.1.2[24] was used to analyze the intercellular interactions within each individual. First, input scRNA-seq data matrix was normalized by TPM (transcripts per million) method and log-transformed with pseudo count of 1. The assigned cell labels were the cell types identified from co-embedding. Ligand-receptor interaction database was CellChatDB v2 excluding non-protein signaling interactions, which finally includes ~ 2300 validated molecular interactions in the analysis. The default parameters were used following the standard CellChat pipeline. Finally, the intercellular communication networks were obtained for each individual and aggregated together for the downstream visualization.

## Identification of candidate pathogenic genes related to signature group G2

We first curated a customized list of 186 genes including all the available cytokines, chemokines, growth factors, NOTCHs, MMPs, and ADMATS with gene expression in this study. The full gene list is shown in **Supplementary Table S9**. For each gene, the maximum gene expression across clusters was taken within each Kmeans group and each individual as input. Then, we identified the universal G2-important genes with mean gene expression across all patients ranked as top 50% and coefficients of variation (CV) less than 2. In total, 63 genes were identified as candidate predictors for the following classification model.

## Classification model construction

To distinguish the controls from At-Risk/ERA patients, we developed a random forest classification model. The input data was gene expression of identified important genes across patients. For each At-

Risk/ERA patient, the maximum gene expression across G2 clusters was taken. For each control, the maximum gene expression across G4 clusters was considered.

The samples were split into train and test subsets at a 7:3 ratio. The R package Caret_6.0.94[43] was used for feature importance evaluation based on recursive elimination algorithm implemented in "rfe" function. Only features with positive importance was kept. Random forest model was trained multiple times with an increasing number of predictors, from the most to least important, using 10-fold cross-validation and repeated 5 times. Each trained model was then evaluated on prediction accuracy on the unseen test set. The above process was repeated 20 times with different random seeds from 1 to 20. The mean and standard deviation of the training and testing accuracy was calculated for each number of predictors.

## Comparison with AMP study

To confirm the expression patterns of newly identified predictors from classification model, we checked the gene expression levels in synovial tissues samples from established RA patients in AMP study[11]. To make it more compatible with cell types in PBMC samples, we only considered 22 clusters defined in original AMP paper that are also present in PBMC populations from 82 synovial tissue samples (Fig. 6A). We collapsed single-cell gene expression profiles into pseudo-bulk count matrices by summing the raw UMI counts for each gene across all cells from the same sample and cluster. For each gene, we normalized counts in each pseudo-bulk sample into counts per million. We averaged the normalized counts across samples, cell types, and genes and visualized the results as heatmaps in Fig. 6A-C respectively.

### Declarations

### Data availability:

scRNA-seq and scATAC-seq data from this paper will be deposited in the GEO database (GSE278746). The output of this study (TF activity heatmap, individual UMAP and cellular network plots) will be available at our Taiji-altra portal (https://wangweilab.shinyapps.io/Taiji_Altra/). All other raw data are available from the corresponding author upon request.

Code availability:

The code to reproduce the data analysis and related figures in this study can be found at https://github.com/Wang-lab-UCSD/Taiji_ALTRA

### Acknowledgments:

This project was supported by grants from the Allen Institute for Immunology and NIH (R01AR065466 to WW and GSF). We thank the study participants for their valuable time and contributions to this study. We thank the clinical research team at University of California, San Diego, University of Colorado, Benaroya Research Institute for recruitment and sample preparation. We thank the Allen Institute founder, P.G. Allen, for his vision, encouragement and support. We thank Adam Savage for support and critical review of the manuscript, the Allen Institute for Immunology operations team for maintaining the productive research environment, and the Human Immune System Explorer (HISE) software development team for their support and dedication. This paper and the research behind it would not have been possible without HISE, a collaborative computational data analysis environment for life sciences research.

### Author contributions:

GSF, WW, KDD, VMH, JHB and TFB conceived and designed the project.

KN, VT, LL, AO, AW, MF, CS, JHB, CS identified and worked with the research subjects who participated and managed the project, with assistance from MLF, MKD, KAK, FZ, LKM, MC, BH, MS.

DB developed methodology and DB supervised the sample collection and processing.

PG, MW, VH, JR performed studies that generated data for the project. LO developed methodology and performed analysis. MAG, PS supervised data acquisition. LB is in charge of project management and TFB for cohort conceptualization.

CL and WW performed bioinformatics analysis with assistance from EBP and PW.

CL, WW, and GSF interpreted analytical results.

CL, WW and GSF drafted the initial manuscript.

All authors reviewed and edited the manuscript. All authors approved the final manuscript.

### Competing interests:

J.H.B. is a Scientific Co-Founder and Scientific Advisory Board member of GentiBio, a consultant for Bristol Myers Squibb and Moderna and has past and current research projects sponsored by Amgen, Bristol Myers Squibb, Janssen, Novo Nordisk, and Pfizer. J.H.B also has a patent for tenascin-C autoantigenic epitopes in rheumatoid arthritis. The other authors declare they have no competing interests. A patent application is being prepared.

## Supplementary Tables

Supplementary Tables S1-S11 are not available with this version.

## Figures

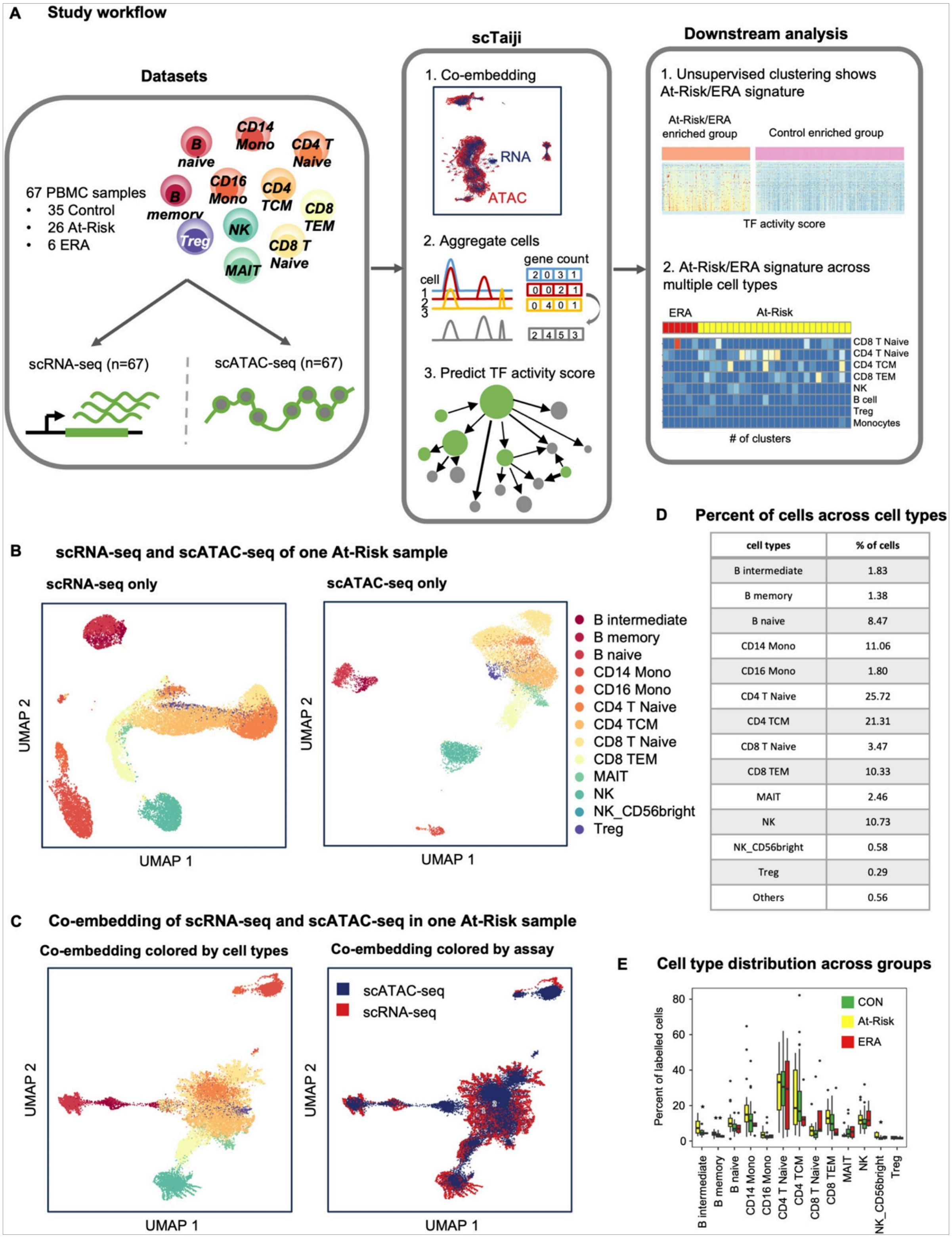

| cell types | % of cells |
|---|---|
| B intermediate | 1.83 |
| B memory | 1.38 |
| B naive | 8.47 |
| CD14 Mono | 11.06 |
| CD16 Mono | 1.80 |
| CD4 T Naive | 25.72 |
| CD4 TCM | 21.31 |
| CD8 T Naive | 3.47 |
| CD8 TEM | 10.33 |
| MAIT | 2.46 |
| NK | 10.73 |
| NK_CD56bright | 0.58 |
| Treg | 0.29 |
| Others | 0.56 |

### Figure 1

Study overview and co-embedding of multi-omics data. (A) Study workflow. PBMC samples including 35 controls (CON), 26 ACPA positive (At-Risk) and 6 early RA (ERA) were utilized for scRNA-seq and scATAC-seq respectively. For each sample, matched data were co-embedded into clusters. Cells in each cluster were aggregated in terms of gene count and open chromatin regions. Then each cluster was used as input of scTaiji to construct a regulatory network and generate the PageRank scores as output.

The following unsupervised clustering revealed At-Risk/ERA signatures that were shared across multiple participants and cell types. (B) UMAP colored by cell types in scRNA-seq cells (left) and scATAC-seq cells (right) respectively for one At-Risk sample. Clusters in both scRNA-seq and scATAC-seq were well separated by cell types. The selected sample represents the typical situation for all the 67 samples. Thirteen cell types include B memory cells, B intermediate cells, B naive cells, CD14 monocytes (CD14 Mono), CD16 monocytes (CD16 Mono), CD4 naive T cells (CD4 T Naive), central memory CD4 T cells (CD4 TCM), CD8 naive T cells (CD8 T Naive), effector memory CD8 T cells (CD8 TEM), mucosal-associated invariant T cells (MAIT cells), natural killer cells (NK), CD56 birght natural killer cells (NK_CD56bright), and regulatory T cells (Treg). (C) UMAP colored by cell types (left) and assays (right) in cells from both scRNA-seq and scATAC-seq for the same sample in Fig. 1B. The color palette of the left plot is the same as Fig. 1B. Blue and red represent scATAC-seq and scRNA-seq. Clusters in co-embedding space were still separated by cell types while scRNA-seq and scATAC-seq cells were well aligned. (D) Percent of total cells across cell types. CD4 Naive and CD4 TCM were the most abundant cell type while B memory cells, CD16 Mono, MAIT, and Treg cells were the relatively rare cell subsets. (E) Cell type distribution across 3 groups of PBMC samples. Yellow, red, green represent At-Risk, ERA, and CON. The color palette is maintained throughout all figures. Centered Log-Ratio (CLR) transformation before Kruskal-Wallis test, *p< 0.1, **p < 0.01. Most cell types showed similar distribution across groups except for B intermediate, B memory, and NK_CD56bright, which were modestly higher in At-Risk compared to other two groups.

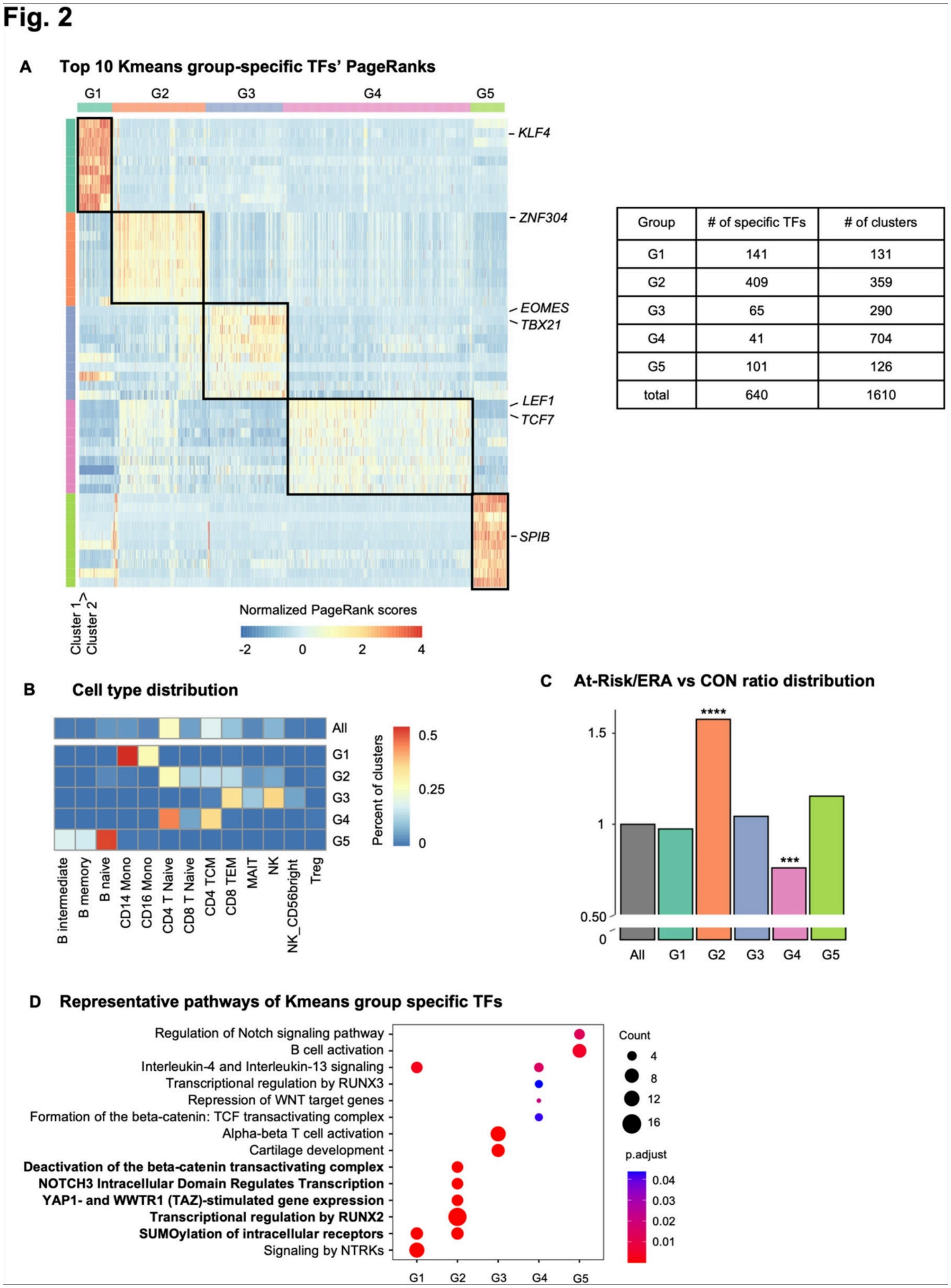

### Figure 2

Unsupervised clustering shows distinct TF regulatory patterns. (A) PageRank scores heatmap of 5 Kmeans group-specific TFs across 1613 clusters. Top 10 TFs from each Kmeans group are selected as rows and colored by their group specificity. Color palette for Kmeans groups is RColorBrewer palette Set2. The color palette is maintained throughout all figures. Clusters in columns are ordered by Kmeans group. Color of the cell indicates the normalized PageRank scores with red displaying high scores. Each

Kmeans group displayed distinct dynamic patterns of TF activity. Side table is the number of the specific TFs for each Kmeans group. G2 has the largest number of specific TFs. (B) Cell type distribution across Kmeans groups. The separate top row represents the overall cell type distribution across all the clusters. The bottom five rows are distributions for five Kmeans groups. Color represents the percentage of clusters of each cell type with red displaying a high percentage. G2 is a multi-lineage group with distribution similar to the overall distribution. Other 4 groups had predominant cell types. (C) At-Risk/ERA vs CON ratio distribution across Kmeans groups. The first gray bar is the overall ratio adjusted to 1 while other bars represent 5 Kmeans groups. G2 is significantly enriched in At-Risk/ERA while G4 is enriched in CON. G1, G3, and G5 show no significant enrichment. (D) Representative Reactome pathways enriched in each Kmeans group-specific TFs. The horizontal axis represents Kmeans groups and the vertical axis represents pathways. Circle size represents the number of TFs in the pathway and color represents the adjusted p-values. Bold text represents signature pathways. G2 exhibits unique enrichment of several RA-related pathways e.g. SUMOylation of intracellular receptors (adjusted p-value < 1e-5), Transcriptional regulation by RUNX2 (adjusted p-value < 1e-5), etc.; Chi-squared test, ***p< 0.001, ****p < 0.0001.

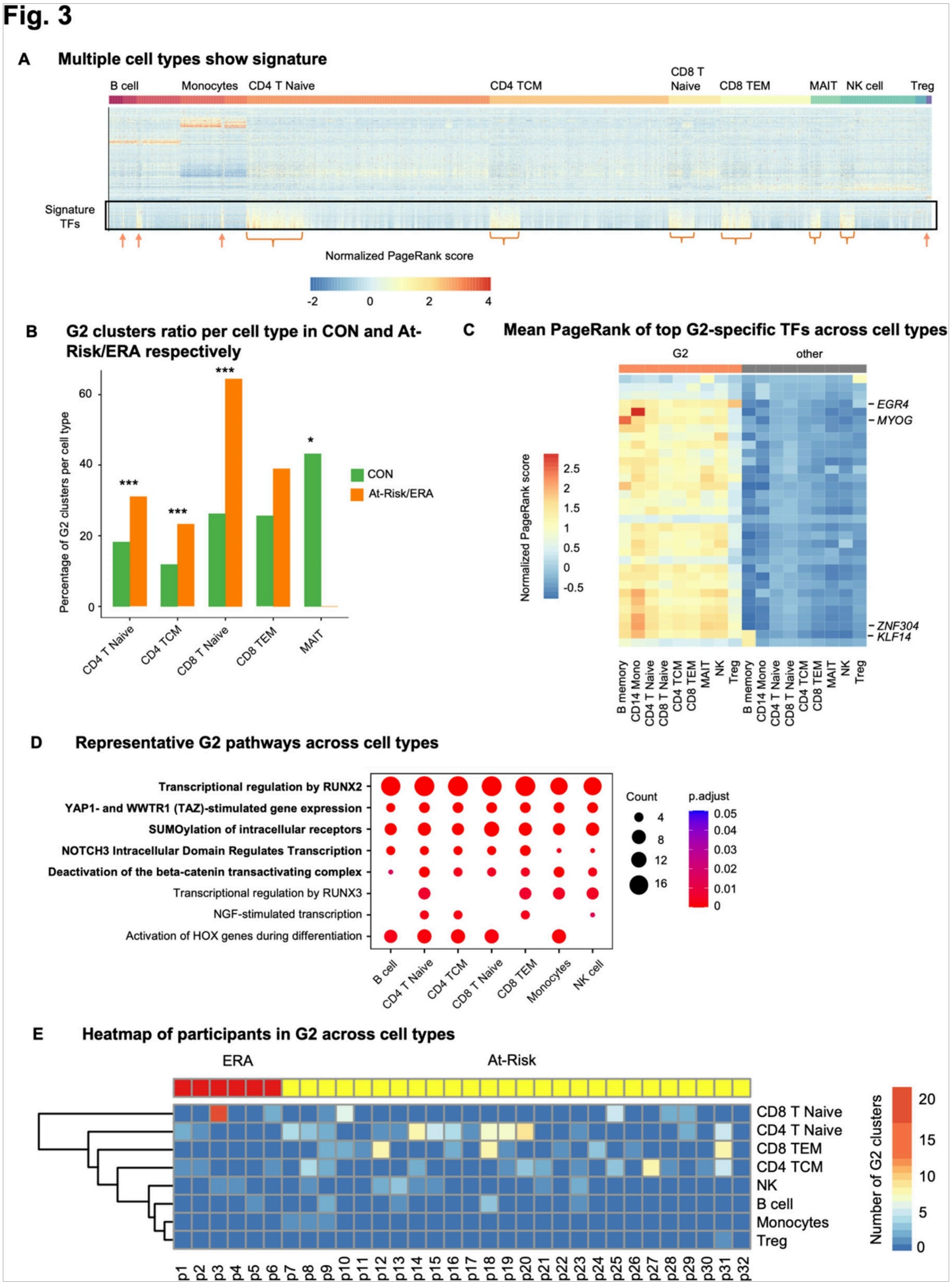

### Figure 3

At-Risk/ERA signature is shared across multiple cell types. (A) Heatmap of PageRank scores of all TFs across all clusters with columns ordered by cell types and Kmeans groups. Color legends are the same as Fig. 2A. The signature TF group is marked by black box. Each cell type displayed high activity in signature TFs. (B) G2 clusters per cell type of the total clusters per cell type in CON and At-Risk/ERA respectively. CD4 T Naive, CD4 TCM, CD8 T Naive, and CD8 TEM are mostly enriched in At-Risk/ERA.

MAIT cells with the signature TFs were only found in CON clusters. (C) Mean PageRank scores of top 50 G2-specific TFs across cell types in G2 and other groups respectively. Rows represent TFs while columns represent cell types in G2 and other groups. Gray represents the average across other 4 groups. Key TFs which are active across almost every cell type are marked by red boxes. (D) Representative enriched pathways of G2-specific TFs across cell types. Bold text represents signature pathways. All the cell types were enriched in signature pathways. (E) Heatmap of At-Risk/ERA participants in G2 across cell types. The horizontal axis shows the individual participants and the vertical axis shows each cell type. Top bar represents the disease states of participants. Color represents the number of clusters per cell type for each participant. All the At-Risk and ERA participants had the signature in at least one cell type but the combination and distribution of cell types are highly variable; Chi-squared test, *p < 0.1, **p < 0.05, ***p<0.01.

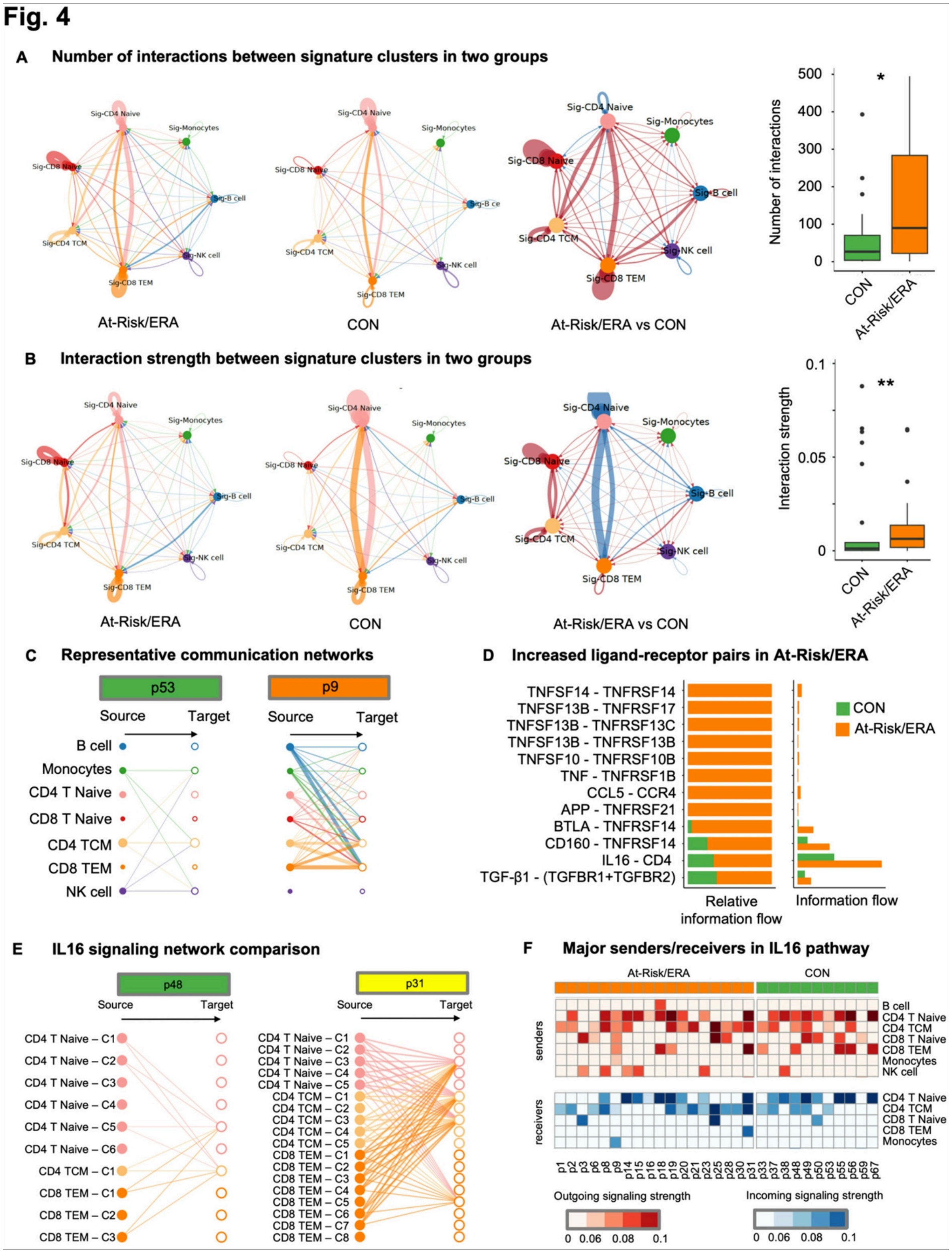

### Figure 4

Distinct cell-cell communication patterns in At-Risk/ERA. (A) Number of cellular interactions within signature clusters in two groups. Edge thickness is proportional to the number of interactions. Thicker edge indicates more interactions. Left and middle circular plots represent networks in At-Risk/ERA and control groups. Color represents the cell type. Right circular plot represents the differential network between At-Risk/ERA and control. Red edge indicates more interactions in At-Risk/ERA and blue is vice versa. Rightmost panel shows the number of interactions in two groups. At-Risk/ERA group has significantly more interactions than control. (B) Interaction strength within signature clusters in two groups. Edge thickness is proportional to the interaction strength. Thicker edge indicates stronger signals. Left and middle circular plots represent networks in At-Risk/ERA and control groups. Color represents the cell type. Right circular plot represents the differential network between At-Risk/ERA and control. Red edge indicates more intense interactions in At-Risk/ERA and blue is vice versa. Rightmost panel shows the interaction strength in two groups. At-Risk/ERA group has significantly stronger interactions than control. (C) Representative cellular communication networks within signature clusters in control and At-Risk patients. Color represents the cell type and thickness of edge weight is proportional to the interaction strength. Thicker edge line indicates stronger signal. Solid and open circles represent source and target respectively. Circle size is proportional to the number of clusters. Both the edge thickness and circle size were normalized and comparable across different networks. At-Risk patient showed much denser and stronger interactions than control across almost all cell types. (D) Increased ligand-receptor pairs in At-Risk/ERA group. The rank is based on the difference in total information flow between At-Risk/ERA and control groups. The total information flow is calculated by summing the probability of all communications between the signature clusters. The left panel showed the relative information flow while the right panel showed the absolute information flow values. (E) Representative IL16 signaling networks within signature clusters in control and At-Risk patients. Each circle represents one Seurat cluster instance with cell type label. Solid and open circles represent source and target respectively. Edge thickness was normalized and comparable across different networks. At-Risk patient showed much denser and stronger interactions than controls. (F) Outgoing and incoming signaling strength of IL16 pathway across cell types in control and At-Risk/ERA groups. The horizontal axis represents the cell types and vertical axis represents each individual, in which IL16 signaling pathway is significant. Gradient red colors represent the total outgoing signaling strength with red displaying higher values. Gradient blue colors represent the total incoming signaling strength with blue displaying higher values.

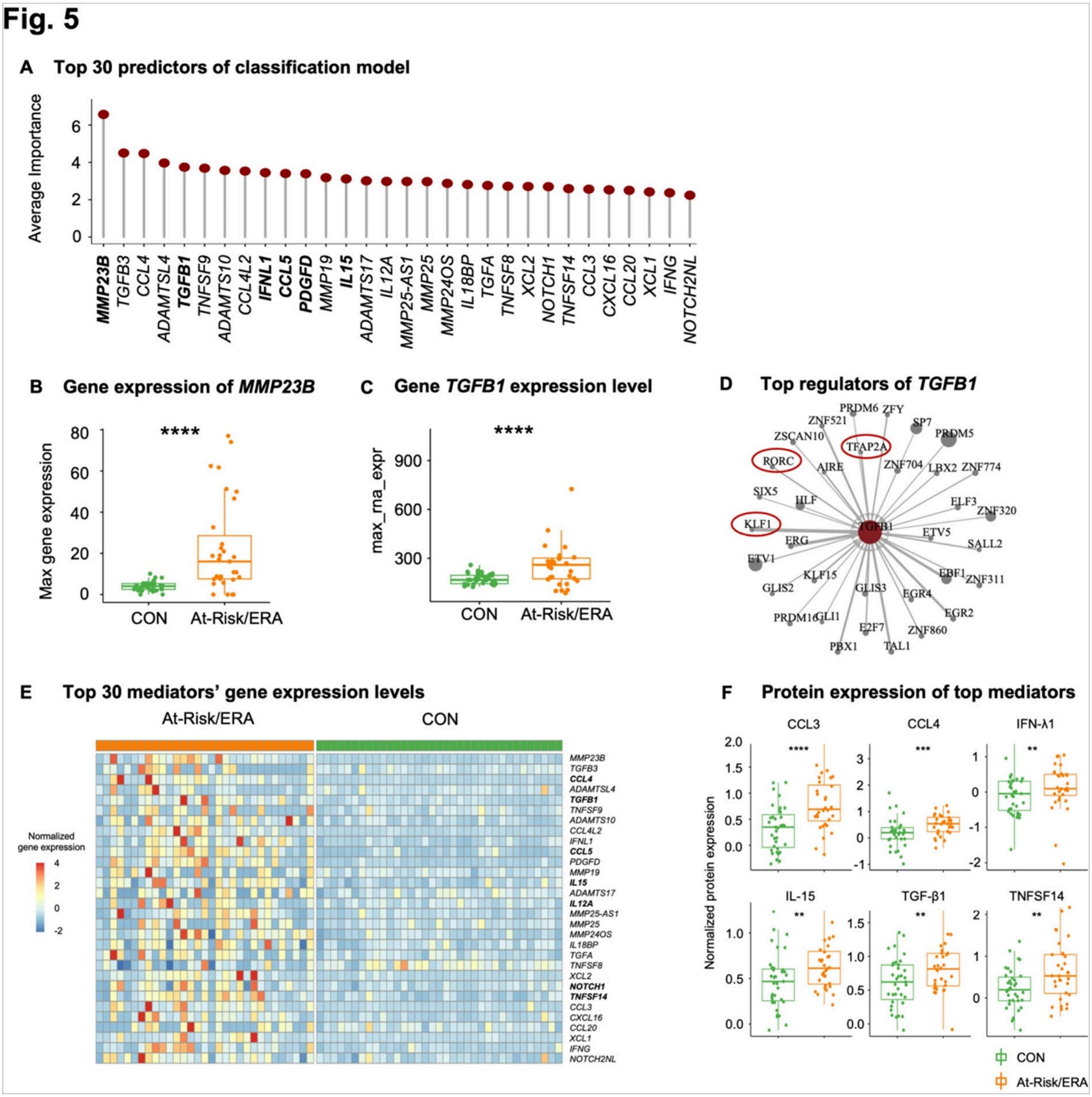

### Figure 5

Identifying key mediators in At-Risk/ERA participants. (A) Top 30 predictors of classification model ranked by the average importance across 20 experiments. Example top predictors include *MMP23B*, *TGFB1*, *IFNL1*, *IL15*, and *CCL5*. (B) Gene expression level of *MMP23B* in each individual. At-Risk/ERA has significantly higher gene expression level than control. (C)Gene expression level of *TGFB1* in each individual. At-Risk/ERA has significantly higher gene expression level than control. (D) Top signature regulators of *TGFB1* in At-Risk/ERA signature clusters. Middle red node is *TGFB1*. Other gray nodes are regulators of *TGFB1*. Gray node size and edge width are proportional to the mean regulatory strength predicted by Taiji. (E) Normalized gene expression of top 30 predictors for At-Risk/ERA and control participants in G2 and G4 clusters respectively. For each gene, the maximum gene expression across clusters was taken within each Kmeans group and each individual. Rows represent mediators while columns represent patients. Red cell represents a higher expression level of the cytokine in the patient. Top 30 predictor cytokines are uniformly more active in At-Risk/ERAs compared to controls. Example genes include *MMP23B*, *CCL4*, *IL12A*, *TNFSF14*, *IL15*, *NOTCH1*, *CCL5*, and *TGFB1.* (F) Protein expression level of six key mediators in each individual. At-Risk/ERA has significantly higher protein expression levels; Wilcoxon rank-sum test, $**p < 0.05$, $***p < 0.01$, $****p < 0.001$.

**Fig. 6**

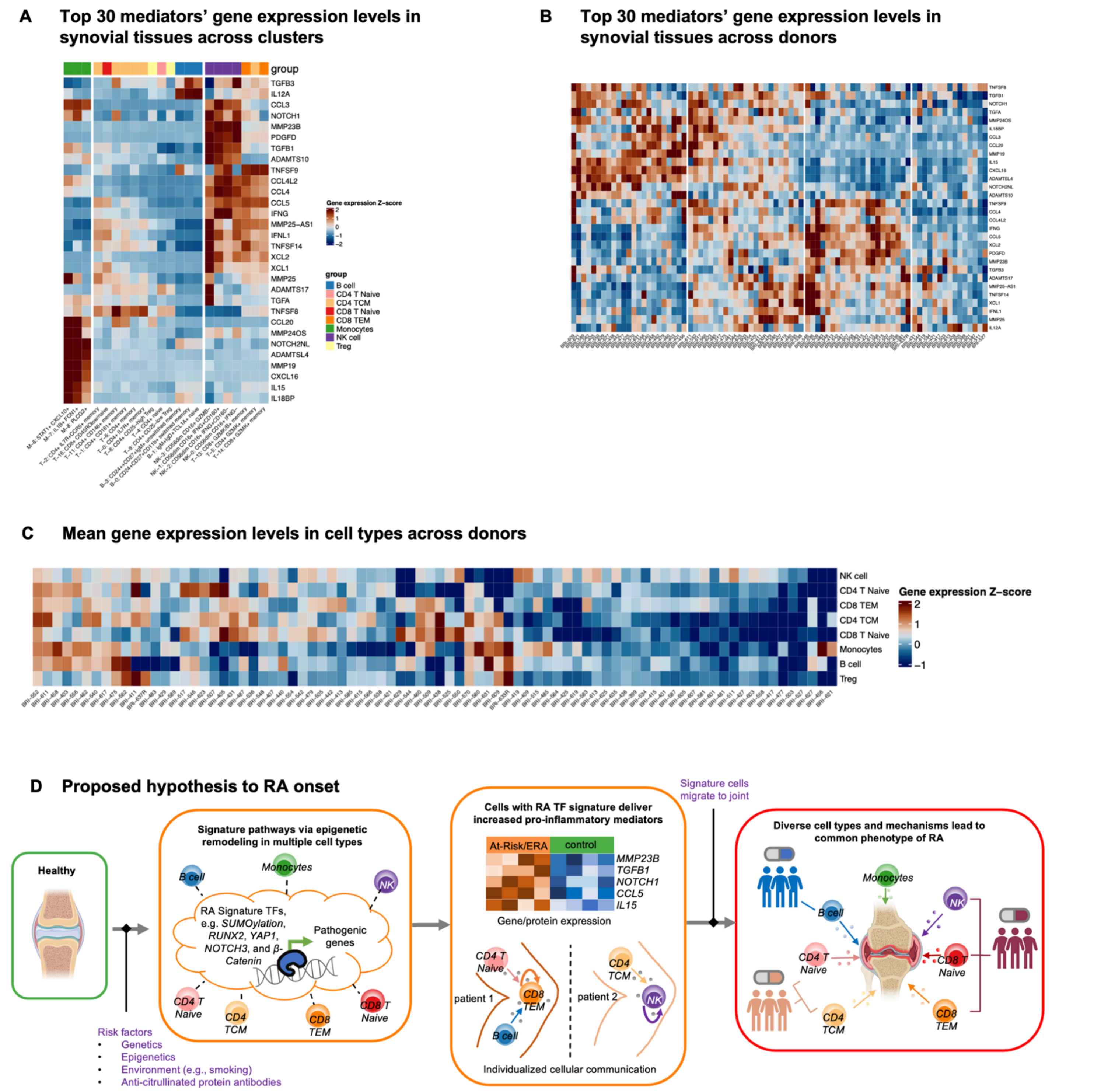

### Figure 6

Gene expression level of identified top mediators in established RA synovial tissues. (A) Heatmap of normalized gene expression of top 30 mediators across 22 pseudo-bulk clusters. Rows represent genes while columns represent pseudo-bulk clusters. Both rows and columns are hierarchically clustered. Color represents the average normalized expression across cells in the cluster, scaled for each gene across clusters. Column annotation legend represents cell types. Top mediators displayed gene expression across multiple cell types. (B) Heatmap of normalized gene expression of top 30 mediators across synovial tissue samples. Rows represent genes while columns represent samples. Both rows and columns are hierarchically clustered. Color represents the average normalized expression across cells in the sample, scaled for each gene across samples. Each sample has its own group of highly expressed genes. (C) Heatmap of normalized gene expression of cell types across samples. Rows represent cell types while columns represent samples. Both rows and columns are hierarchically clustered. Color represents the average normalized expression across 30 mediators, scaled for each cell type across samples. Each sample has its own combinations of dominant cell types expressing the top mediators. (D) Proposed hypothesis to RA onset. Under the influence of risk factors such as genetics and environmental exposures, epigenetic remodeling took place in multiple cell types involving signature pathways like SUMOylation, RUNX2, YAP1, NOTCH3, and β-Catenin Pathways. The signature TFs drive a characteristic set of pro-inflammatory genes in receiver cells that can, in turn, contribute to the onset and perpetuation of RA. Diverse cell types and pathogenic mechanisms can drive a common clinical phenotype known as RA and could explain the wide variation in clinical response to agents that target individual cytokines or cell types.

### Supplementary Files

This is a list of supplementary files associated with this preprint. Click to download.

- Supplementarymaterialandlegends.docx
